# Shining a Light on Prostate Cancer: Photodynamic Therapy and Combination Approaches

**DOI:** 10.3390/pharmaceutics15061767

**Published:** 2023-06-19

**Authors:** Hicham Wahnou, Ibtissam Youlyouz-Marfak, Bertrand Liagre, Vincent Sol, Mounia Oudghiri, Raphaël Emmanuel Duval, Youness Limami

**Affiliations:** 1 Laboratory of Immunology and Biodiversity, Faculty of Sciences Ain Chock, Hassan II University, B.P. 2693, Maarif, Casablanca 20100, Morocco; hwwahnou@gmail.com (H.W.); mounia.oudghiri@univh2c.ma (M.O.); 2 Laboratory of Health Sciences and Technologies, Higher Institute of Health Sciences, Hassan First University of Settat, Settat 26000, Morocco; ibtissam.marfak@uhp.ac.ma; 3 Univ. Limoges, LABCiS, UR 22722, F-87000 Limoges, France; bertrand.liagre@unilim.fr (B.L.); vincent.sol@unilim.fr (V.S.); 4 Université de Lorraine, CNRS, L2CM, F-54000 Nancy, France

**Keywords:** photodynamic therapy, prostate cancer, reactive oxygen species, cell death, immune response, tumor vascular damage, clinical trials, combination approaches

## Abstract

Prostate cancer is a major health concern worldwide, and current treatments, such as surgery, radiation therapy, and chemotherapy, are associated with significant side effects and limitations. Photodynamic therapy (PDT) is a promising alternative that has the potential to provide a minimally invasive and highly targeted approach to treating prostate cancer. PDT involves the use of photosensitizers (PSs) that are activated by light to produce reactive oxygen species (ROS), which can induce tumor cell death. There are two main types of PSs: synthetic and natural. Synthetic PSs are classified into four generations based on their structural and photophysical properties, while natural PSs are derived from plant and bacterial sources. Combining PDT with other therapies, such as photothermal therapy (PTT), photoimmunotherapy (PIT), and chemotherapy (CT), is also being explored as a way to improve its efficacy. This review provides an overview of conventional treatments for prostate cancer, the underlying principles of PDT, and the different types of PSs used in PDT as well as ongoing clinical studies. It also discusses the various forms of combination therapy being explored in the context of PDT for prostate cancer, as well as the challenges and opportunities associated with this approach. Overall, PDT has the potential to provide a more effective and less invasive treatment option for prostate cancer, and ongoing research is aimed at improving its selectivity and efficacy in clinical settings.

## 1. Introduction

With over 1.4 million new cases and more than 375,000 deaths worldwide in 2020, prostate cancer is a major public health concern [1]. Prostate cancer is the second most commonly occurring cancer in men and the fourth most common cancer overall. In addition, the rising incidences of prostate cancer in the last decade are concerning, even if it is likely attributed to earlier detection through increased screening [1]. Conventional treatment options such as surgery, radiation therapy, hormone therapy, chemotherapy, and immunotherapy have limitations, including high invasiveness, poor specificity, and adverse effects. In this context, PDT has emerged as an innovative and promising treatment option for prostate cancer due to its high specificity, low invasiveness, and reproducibility [2,3].

PDT involves the use of photosensitizing agents and light to activate them, leading to localized cancer cell death [2]. However, PDT alone may not be sufficient to eradicate prostate cancer completely. Thus, the combination with other therapeutic options may provide opportunities to draw on the strong points of each option and offset their weaknesses, leading to additive or synergistic therapeutic effects [4].

This review article aims to explore the potential of PDT as a treatment for prostate cancer, including its mechanism of action, limitations, photosensitizers, and combination with other therapies. It also provides insights into the future directions of PDT in the context of prostate cancer treatment.

To ensure the accuracy and comprehensiveness of our review, our team embarked on a rigorous data collection and search process from a wide range of reputable databases, including Google Scholar, Pubmed, Springer, Elsevier ScienceDirect, and Web of Science. We focused on scrutinizing studies published from 2000 to 2023 and we included one older study for contextualization. In order to maintain consistency and ensure a thorough analysis, only articles containing English texts were selected, and we conducted keyword and heading searches for the following terms: prostate cancer, photodynamic therapy, natural photosensitizers, synthetic photosensitizers, prostate cancer treatment complications, and combination cancer therapies.

Then, a thorough selection process was conducted, starting with 2101 records. Before proceeding to screen full-text documents, duplicate papers and irrelevant works were eliminated. The inclusion criteria were strict and consisted of original articles, review papers, book chapters or websites that met specific factors, thus ensuring the accuracy and quality of the information presented in this paper. Overall, 96 references were selected for our study, which included articles or websites specifically dedicated to providing context, epidemiological data, and reports of clinical studies.

## 2. Conventional Treatments for Prostate Cancer and Complications

### 2.1. Surgery

Surgery is a common treatment option for prostate cancer and involves the partial or radical removal of the prostate gland. This procedure, known as a prostatectomy, can be performed using various techniques [5]. Nevertheless, many potential complications may arise in this procedure, such as incontinence and erectile dysfunction resulting from surgical damage to the urinary sphincter and erectile nerves [6]. These adverse effects can greatly impact a patient’s quality of life and are important considerations in prostate cancer treatment decision-making [6].

### 2.2. Radiation Therapy

Radiation therapy is a cancer treatment that uses high-energy X-rays or other types of radiation to eliminate cancer cells. Two types of radiation therapy are used: external and internal [5]. External radiation therapy involves using a machine to direct radiation toward the cancer site, while internal radiation therapy places a radioactive substance directly into or near the cancer site [5,7]. Radiation therapy for prostate cancer increases the risk of developing bladder and/or gastrointestinal cancer. Furthermore, a higher rate of long-term complications, such as impotence and urinary problems, can also occur [5,8].

### 2.3. Hormone Therapy

Hormone therapy, also known as androgen deprivation therapy (ADT), is a treatment that aims to remove or block male hormones, which can cause cancer cells to grow. This can be achieved through drugs such as abiraterone acetate and luteinizing hormone-releasing hormone agonists, or surgery to remove the testicles (orchiectomy), or the use of antiandrogens that block the action of male hormones [5]. Hormone therapy can help shrink the tumor and slow the growth of cancer, but it may also cause side effects including hot flashes, loss of libido, and weakened bones [5,7].

### 2.4. Chemotherapy

Chemotherapy is a treatment that uses powerful drugs to destroy cancer cells. In prostate cancer, chemotherapy is usually reserved for advanced cases that no longer respond to hormone therapy [5]. Chemotherapy can cause side effects such as hair loss, nausea, vomiting, fatigue, and an increased risk of infection due to a weakened immune system. However, these side effects can be managed with medications and other supportive therapies [7,9].

### 2.5. Cellular Immunotherapy

Immunotherapy is a cancer treatment that harnesses the patient’s own immune system to attack cancer cells. It involves the use of substances or cells, either naturally produced by the body or created in a lab, to enhance or restore the body’s natural defenses against cancer [5]. In the case of prostate cancer, Sipuleucel-T, an autologous cellular immunological agent, is intended for patients with metastatic hormone-refractory prostate cancer. It consists of exposing the patient’s peripheral blood mononuclear cells to a recombinant fusion protein composed of prostatic acid phosphatase, an antigen that is highly expressed in most prostate cancer cells, and a granulocyte-macrophage colony-stimulating factor. The mechanism of action is not fully elucidated, but it is likely to work through antigen-presenting cells (APCs) to stimulate a T-cell immune response [10]. The most reported side effects, including chills, fever, headache, myalgia, sweating, and influenza-like symptoms, were attributed to cytokine release usually within the first 24 h of infusion. However, no increased risk of autoimmune complications has been reported to date [5].

### 2.6. Other Therapeutic Approaches

Prostate cancer treatment has come a long way, with several innovative therapies reported in clinical studies [5]. Cryosurgery, or cryotherapy, involves the use of an instrument to freeze and destroy cancerous prostate cells. High-intensity-focused ultrasound therapy, on the other hand, uses ultrasound waves to break down cancer cells. Additionally, proton beam radiation therapy employs tiny, positively charged particles to target and kill tumor cells [5].

Besides these therapeutic approaches, another promising treatment for prostate cancer is PDT. The principle of this alternative treatment is the use of a photoactivated drug to selectively destroy cancer cells while minimizing damage to healthy tissues and side effects.

## 3. PDT

### 3.1. Mechanism of Action

**Figure 1:**
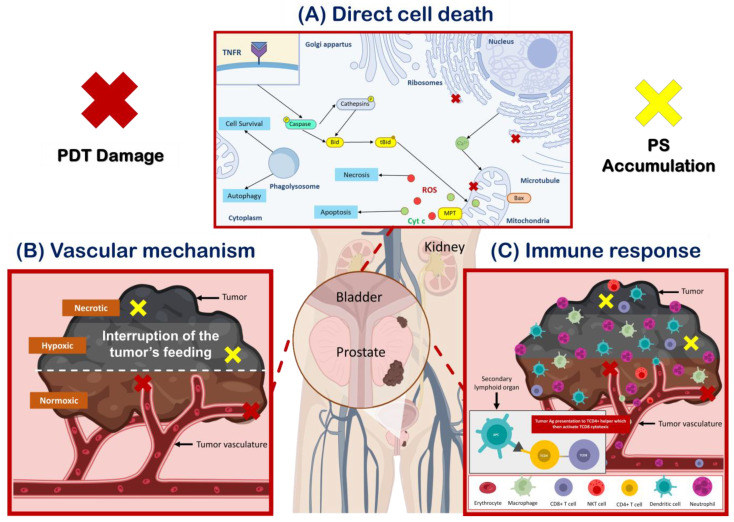
PDT destroys tumor cells by three mechanisms of action. (**A**) Induction of cell death through various pathways depends on the location of the photosensitizer (PS) and the extent of damage to the organelles. Mitochondrial damage caused by PDT results in the loss of membrane permeability and the release of pro-apoptotic mediators, while damage to the endoplasmic reticulum (ER) leads to the release of cellular calcium deposits. Lysosomal damage, on the other hand, causes the release of proteolytic enzymes upon illumination, and can also trigger autophagy. In cases where apoptosis is dysfunctional, necrosis and autophagy may be the primary modes of cell death after PDT. It is important to note that multiple PSs may localize in multiple organelles, and therefore, simultaneous activation of death pathways may occur (adapted from Mroz, P et al. [19]). (**B**) PDT can induce vascular damage, leading to the interruption of the tumor’s feeding causing its death through various mechanisms. (**C**) PDT can also activate innate and adaptative immunity, leading to a systemic antitumor immune response and the destruction of tumor cells.

PDT uses a photosensitizer (PS), molecular oxygen, and light to generate reactive oxygen species (ROS) that can trigger tumor cell death [11]. PDT can cause tumor destruction by three mechanisms: direct cell death, tumor vascular damage, and an immune response (Figure 1) [11,12].

Direct cell death (Figure 1A) occurs through both programmed cell death (apoptosis) and non-programmed cell death (necrosis) pathways [13]. Necrosis is characterized by rapid cell death leading to the release of cellular components and molecules that promote inflammation, while apoptosis is a genetically encoded, energy-dependent process that can initiate an immune response [14,15]. PDT can also induce unconventional modes of cell death in cancer cells, including paraptosis, parthanatos, mitotic catastrophe, pyroptosis, necroptosis, and ferroptosis [13]. In addition, a growing body of evidence suggests that PDT can also trigger immunogenic cell death (ICD) [16], which has emerged as a promising strategy for eliminating tumor cells by promoting T-cell adaptive immune responses and inducing durable immunological memory [13,16,17].

PDT can also damage the tumor microvasculature (Figure 1B), leading to the interruption of the tumor’s feeding and, consequently, to the death of the tumor cells. This vascular mechanism is achieved by concentrating the PS in the vascular system and using a short drug-light interval [18]. PDT vascular effect can be selectively applied to the tumor and surrounding healthy tissue, with important advantages over PDT protocols that require PS accumulation in the tumor cells [17,18].

Finally, PDT can induce an immune response (Figure 1C) that can contribute to long-term tumor control [18]. PDT has been shown to influence the adaptive immune response through either stimulation or suppression, depending on the treatment protocol [6]. The oxidative damage inflicted by PDT on the tumor stroma triggers an acute inflammatory response initiated by the release of pro-inflammatory mediators. These mediators attract the host’s innate immune cells, which can activate a systemic antitumor immune response. PDT-induced necrosis of tumors and their vasculature can activate CD8 cytotoxic T lymphocytes that can specifically destroy tumor cells and circulate throughout the body for long periods [18].

### 3.2. Photosensitizers (PSs)

PSs are molecules that can absorb light and transfer the absorbed energy to the surrounding molecules (e.g., oxygen), leading to the generation of reactive oxygen species (ROS) that can cause cell death. These PSs are generally divided into two categories: tetrapyrrolic macrocycles (i.e., porphyrins, chlorins, bacteriochlorins, phthalocyanines, and their derivates) and no tetrapyrrolic macrocycles (i.e., methylene blue, curcumin, phenalenon, coumarin, anthraquinone, boron-dipyrromethene florophore (bodipy), and their derivatives) [20,21,22,23]. Tetrapyrrolic macrocycles are the most widely used PSs in anticancer PDT and, among them, porphyrins are the most studied. Thus, porphyrin-derived PSs are further classified into three generations [24].

The first-generation PS, such as the hematoporphyrin derivative (HpD) and Photofrin^®^, were the first photosensitizers to be used clinically in PDT. However, these molecules have some drawbacks, such as a mixture of PSs, prolonged skin photosensitization, no selectivity, and suboptimal tissue penetration [25].

Second-generation PSs were developed to address these issues. These compounds are chemically pure, absorb light at a longer wavelength, and cause significantly less skin photosensitization post-treatment. They must also be at least as efficient in eradicating tumors as Photofrin^®^, which is currently the gold standard for PDT [26].

Third-generation PSs, which are bound to targeting agents (antibodies, sugar, folic acid, polyamine, peptide, etc.), are able to be selectively accumulated within tumor tissues and represent an active research area in the field [27,28,29,30,31,32,33,34,35].

Of note, fourth-generation PSs are still in the early stages of development and are characterized by their ability to be associated with nanoparticles and/or activated by specific stimuli, such as light at a specific wavelength, changes in pH or temperature, ultrasound, or X-rays [36]. This allows for even greater control over the location and timing of the PS activation, potentially reducing side effects and improving treatment efficacy. Fourth-generation PSs are still a relatively new area of research but have shown promising results in preclinical studies [37,38,39,40].

### 3.3. Natural and Synthetic PS

Herbal medicines have been used for centuries to treat various human ailments, and many modern medicines are derived from medicinal plants. There is a growing interest in using natural products, including herbal extracts and compounds, as possible lead components in cancer drug discoveries. Natural products are a valuable source for the development of drugs and the discovery of new PSs [41,42,43,44]. In recent decades, efforts have been made to isolate new products of natural origin from microbes, plants, and other living organisms, leading to the discovery of a number of anti-cancer drugs [41]. Researchers have found new active chemical compounds in natural extracts that display efficient PS properties, and numerous compounds have already been identified as potential candidates to be used as PSs [44]. Furthermore, screening studies of natural extracts have identified new PSs with cyclic tetrapyrrole structures [45]. Additionally, in a 2022 review published by Kubrak TP et al., various photochemicals were described for their light-absorbing properties and their anticancer activity including furanocoumarins, polyacetylenes, thiophenes, curcumins, alkaloids, and anthraquinones [20].

On the other hand, synthetic PSs are those that are created in the laboratory through chemical synthesis. These PSs are designed to have specific properties that allow them to effectively target cancer cells and destroy them upon exposure to light. They are often optimized to have higher light absorption at longer wavelengths, improved tissue penetration, and reduced toxicity compared to earlier generations of PSs [37,46]. Synthetic PSs can be produced in large quantities and with a high degree of purity, making them a reliable and cost-effective option for PDT. Some examples of synthetic PSs include benzoporphyrins, texaphyrins, purpurin, chlorin e6, bacteriochlorin. and pyropheophorbide.

**Figure 2:**
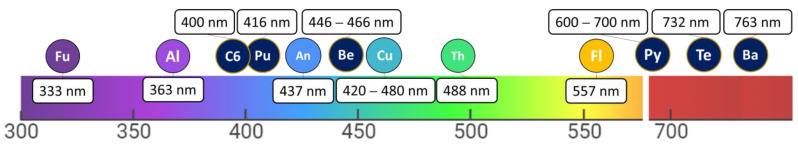
Absorption wavelengths of non-tetrapyrrolic PS derivatives (furanocoumarins (Fu), thiophenes (Th), curcumins (Cu), flavonoids (Fl), anthraquinones (An), alkaloids (Al)) and tetrapyrrolic PS derivatives (benzoporphyrin (Be), texaphyrins (Te), purpurin (Pu), chlorin e6 (C6), bacteriochlorin (Ba), pyropheophorbide (Py)).

Figure 2 presents some natural and synthetic PSs according to their absorption wavelengths.

**Figure 3:**
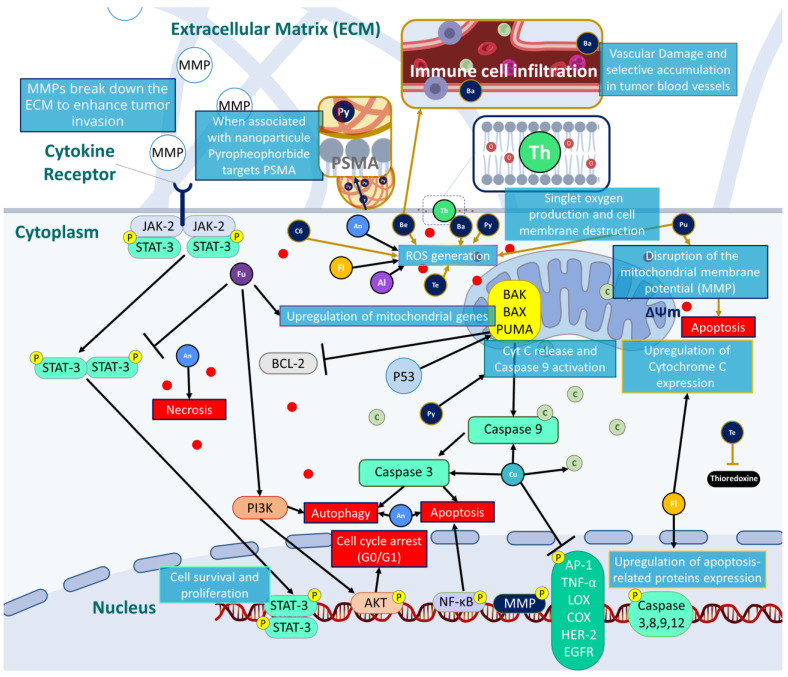
Possible mechanisms of action to induce prostate cancer eradication by targeting several pathways involving oxidative stress, cell death and cell cycle arrest.

An updated summary of natural and synthetic PSs and their potential mechanism of action to target prostate cancer are presented in Table 1 and Figure 3.

### 3.4. Ongoing Clinical Studies in Prostate Cancer

In clinical settings, the implementation of PDT for prostate cancer involves several key steps. First, a photosensitizing agent is administered to the patient, either systemically or directly into the tumor site. The choice of photosensitizer depends on factors such as its absorption and distribution properties, as well as its ability to selectively accumulate in tumor tissues [74]. Once the photosensitizer has been adequately distributed, a specific light source is selected, considering factors, such as wavelength and power output [74]. Light delivery techniques may vary, including intraluminal or interstitial illumination [75]. For instance, intraluminal light sources can be used for prostate cancer by inserting light-emitting devices through catheters or endoscopes. Interstitial PDT involves the placement of optical fibers or diffusers directly into the prostate tissue, allowing for light delivery to deeper tissue layers [75]. These techniques aim to achieve optimal light distribution and tumor coverage.

PDT is a promising treatment option for prostate cancer, with the potential to provide a safe and effective alternative to conventional therapies. However, in order to fully evaluate the safety and efficacy of PDT for prostate cancer, clinical trials are needed. These trials can provide valuable insights into the effectiveness of PDT, as well as help to identify any potential side effects or limitations of the treatment.

To date, several clinical trials have been conducted on the use of PDT for prostate cancer, with promising results.

By conducting research on clinical trials to further explore the potential of PDT for the treatment of prostate cancer [76], we found that out of the 14 studies conducted (Table 2), using the keywords “Photodynamic therapy” and “Prostate cancer”, only one study posted their results [77].

### 3.5. Multimodal Synergistic Therapies to Overcome PDT Limitations

**Figure 4:**
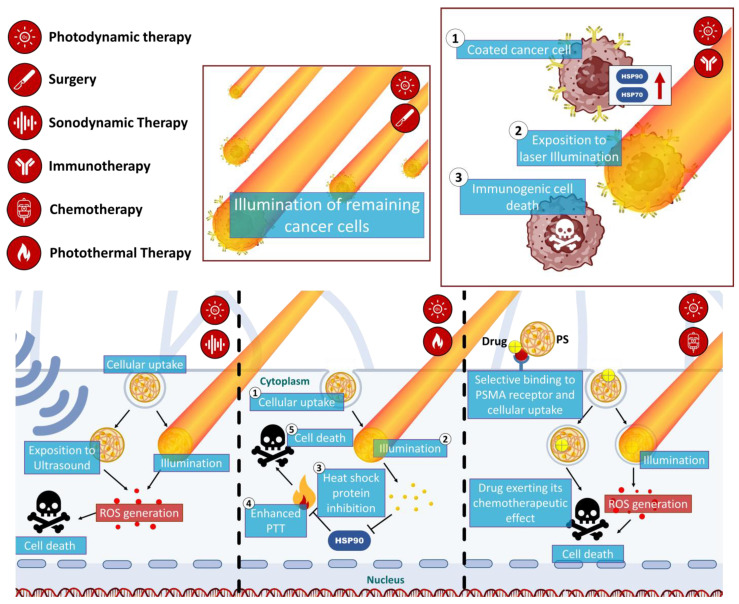
Schematic illustration of mechanisms of the multimodal synergistic therapies targeting prostate cancer using PDT.

Combination approaches in PDT for cancer treatment address limitations related to tumor size and location [78]. Larger tumors and tumors located in challenging anatomical areas can pose difficulties in achieving complete eradication with PDT alone due to the limited light penetration depth [79]. However, by combining PDT with other treatment modalities, such as surgery, sonodynamic therapy, photoimmunotherapy, chemotherapy, and photothermal therapy, these limitations can be overcome [80]. For larger tumors, combination approaches can sensitize the tumor or target residual cancer cells, thus enhancing the therapeutic effect. Multimodal therapies that combine PDT with other treatments have shown promise in the treatment of multidrug-resistant and hypoxia-related prostate cancers (Figure 4) [4,81]. However, challenges arise in terms of complexity, potential side effects and the requirement for rigorous testing of optimal sequencing and timing strategies.

#### 3.5.1. PDT/Surgery

Surgery is a common treatment for prostate cancer, but it is not always successful in eradicating all cancer cells. Recent studies have explored the potential benefits of combining surgery with PDT in the treatment of prostate cancer. A retrospective study published in 2019 by Pierrard V et al. [82] investigated the safety and effectiveness of salvage radical prostatectomy (surgical removal of the prostate gland) after vascular-targeted photodynamic therapy (VTP) treatment. The study included 45 patients who underwent a salvage prostatectomy followed by VTP salvage due to persistent or recurrent cancer. The results showed that the combination was a safe and effective treatment option for localized prostate cancer. The study also reported that the combination therapy did not result in any severe complications or adverse events [82]. Furthermore, the feasibility, safety, and effectiveness of this combination in treating most locally recurrent prostate cancer were also reported by a previous study where various outcomes were reported including operation time, blood loss, complications, urethral catheterization time, functional outcomes, and short-term oncologic outcomes [83].

#### 3.5.2. PDT/Sonodynamic Therapy

Although there are no specific studies on the use of PDT and sonodynamic therapy (SDT) for prostate cancer treatment, these therapies are currently being studied for their potential in cancer treatments [39,84]. PDT and SDT utilize sensitizers that can produce ROS, which can cause damage to cancer cell membranes. The combination of these therapies has demonstrated promising results in preclinical studies for various types of cancer, such as brain cancer [39]. In fact, in the prostate cancer context, the penetrating power of ultrasound avoids the use of an endoscope (and light) in the bladder while allowing the activation of the PS [84]. Ongoing research aims to identify effective sensitizers and optimal delivery methods to enhance the effectiveness of PDT and SDT in prostate cancer treatments. While further studies are necessary to determine the safety and efficacy of these therapies for prostate cancer, the potential benefits of PDT and SDT offer a promising avenue for the development of effective cancer treatments.

#### 3.5.3. PDT/Photoimmunotherapy

Photoimmunotherapy (PIT) is an innovative treatment that harnesses the power of phototherapy and immunotherapy to combat cancer by inducing antitumor immune responses and preventing cancer recurrence [85]. Among the different approaches to PIT, one of the most promising and rapidly expanding is near-infrared (NIR)-PIT. This approach selectively and locally destroys cancer cells by releasing damage-associated molecular patterns (DAMPs) and tumor-associated antigens (TAAs), which trigger an antitumor immune response. NIR-PIT has demonstrated remarkable results in preclinical and clinical applications for various types of cancer including prostate cancer [85,86]. In fact, in a study using a prostate-specific membrane antigen (PSMA)-expressing a prostate cancer cell line, NIR-PIT with a fully human IgG1 anti-PSMA monoclonal antibody (mAb) conjugated to a specific photoabsorber resulted in specific binding and cell-specific killing after exposure to NIR light [86].

#### 3.5.4. PDT/Chemotherapy

In the last few years, researchers have developed a new treatment strategy for prostate cancer known as photochemotherapy. This approach combines the benefits of PDT and chemotherapy to deliver cancer-fighting drugs selectively to tumor cells. Unlike traditional chemotherapy, photochemotherapy can target cancerous tissues without damaging surrounding healthy cells, potentially reducing unwanted side effects. By combining these two therapies, the effectiveness of the treatment is enhanced, with chemotherapy compensating for the limited light penetration in the PDT. Furthermore, chemotherapy may increase the sensitivity of cancer cells to ROS or hyperthermia, resulting in a synergistic effect [87]. Furthermore, in vivo studies using athymic nude mice and BALB/c nude mice, have also demonstrated the effectiveness of photochemotherapy in treating prostate cancer [88,89].

#### 3.5.5. PDT/Photothermal Therapy

Photothermal therapy (PTT) is a form of phototherapy that uses light energy to produce heat energy, which can kill cancer cells [4]. PTT has been used to treat prostate cancer by irradiating a photothermal agent with an external light source of a specific wavelength, typically NIR light. PTT has been combined with PDT to enhance the effects of both therapies. The thermal effect of PTT can improve local blood flow and increase the oxygen concentration in the tumor tissue, making up for the deficiency of PDT in a hypoxic environment [90]. Many studies have reported the combination of PDT and PTT using nanomaterials as drug carriers [4,90]. Some studies have used self-assembled nanoparticles, such as melanin-like polydopamine nanoparticles, to deliver photothermal and photodynamic agents for synergistic therapy [4,90]. However, the low photoconversion efficiency of some materials used in these therapies raises issues about how to maximize the synergistic effect of PDT and PTT.

## 4. Conclusions

In the last decades, steady progress in prostate cancer patients’ care has been noted due to the broad application of early detection and progress in therapeutic approaches. In this context, PDT is a promising approach to treat prostate cancer that has the potential to provide a minimally invasive, highly targeted approach and negligible side effects on normal tissues when compared to conventional therapies. Several PDT approaches have already been approved by regulatory authorities including the FDA and the European Medicines Agency. Furthermore, combining PDT with other therapy modalities such as chemotherapy, radiotherapy, surgery, and immunotherapy seems to be more effective against tumor growth. Advances in therapeutic approaches to develop innovative organic PSs for enhanced PDT are being reported worldwide [91]. Recently, PDT has entered the era of vectorization and encapsulation into drug delivery systems using innovative strategies to increase the delivery of PSs to tumor tissues [89,92,93,94,95]. Furthermore, several research studies reported the use of PDT to treat different cancer types, including colorectal cancer, melanoma, and glioblastoma as well as other diseases such as rheumatoid arthritis [92,93,94,95,96,97]. Despite considerable progress in understanding the underlying mechanisms of photodynamic therapy, further clinical studies are still needed to improve its modalities.

## Figures and Tables

**Table 1:** Natural and Synthetic PSs and their potential mechanism of action in PDT of prostate cancer (adapted from Muniyandi K et al. [43]).

**Natural PSs**				
**Name**	**Natural Sources**	Type of PDT *	**Mechanism of Action**	
Furanocoumarins (Fu)	*Ruta graveolens*, *Angelicae dahuricae*, *Glehnia littoralis*, *Syzygium Sps Tetradium daniellii*, and *Ficus sps*.	Type II	- Inhibition of STAT-3 pathway and PI3K/AKT pathway [47,48], - Activation of p53 [49], - Forming adducts with DNA and cell cycle arrest [50], - Upregulation of the mitochondrial genes BAK, BAX, and PUMA [49,51].	
Thiophenes (Th)	*Echinops*, *Eclipta*, *Pluchea*, *Artemisia*, *Atractylodes*, *Tagetes*, *Porophyllum*, and *Xanthium*.	Type I and II	- Accumulation in fatty acid-rich regions and by producing single oxygen leading to cell membrane destruction [52].	
Curcumins (Cu)	*Curcuma longa (*family *Zingiberaceae*, species *Curcuma)*	Type I	- Reduction of matrix metalloproteases’ (MMPs) enzyme production [53], - Downregulation of inflammatory pathways molecules (e.g., tumor necrosis factor (TNF)-α, lipoxygenase (LOX), and cyclooxygenase (COX)-2) transcription factors (e.g., AP-1, nuclear factor (NF)-κB) and growth factors (e.g., epidermal growth factor (EGFR) and HER-2) [54,55], - Activation of caspase-3, -8, and -9 and cytochrome c release [56], - Angiogenesis inhibition [44,54,55,57].	
Flavonoids (Fl)	Herbs, Vegetables, Seeds, Cereals, Fruits, Flowers, and Nuts	Type II	- ROS generation, DNA damage [58], - Upregulate the expression of apoptosis-related proteins [58,59], - Increase the expression and release of cytochrome c [59].	
Anthraquinones (An)	*Polygonum cuspidatum*, *H. lycioides*, *Aloe vera*, *Rheum palmatu*, *Polyathia suberosa*, *Xanthoria parietina*, *Ramularia collo-cygni.* and *H. perforatum*	Type I and II	- ROS generation [60], - Induction of apoptosis, necrosis, or autophagy [60,61].	
Alkaloids (Al)	*Guatteria blepharophylla*, *Berberis vulgaris*, *Sanguinaria Canadensis*, *Peganum harmala*, and *Indigofera tinctoria*.	Type I	- ROS generation [62], - Promotion of apoptosis and autophagy by regulating several genes (Bax, Bcl-2, Bcl-xL, NF-κB) and various caspase proteins [62,63,64].	
**Synthetic PSs**				
**Name**	**Commercial Name/Excitation Wavelength**	**PS Class/Source**	Type of PDT *	**Mechanism of Action**
Benzoporphyrin (Be)	Visudyne λ = 689 ± 3 nm	Porphyrin-based PS (Derived from benzoporphyrin)	Type II	- Lymphocyte infiltration and inhibition of tumor metastasis [26,65], - Once excited, it enhances the formation of ·OH from intracellular H_2_O_2_ via a Fenton-like reaction by a catalyzer [26].
Texaphyrins (Te)	Lutrin, Antrin, Optrin, Xcytrin λ = 732 nm	Texaphyrin-based PS (Derived from expanded porphyrins)	Type I	- Weakens the cellular antioxidant system by inhibiting thioredoxin reductase and by oxidizing reducing species (e.g., ascorbate), leading to a buildup of damaging ROS (e.g., peroxide) [46,66].
Purpurin (Pu)	Purlytin λ = 664 nm	Porphyrin-based PS (Derived from chlorophyll)	Type II	- Production of singlet oxygen and other ROS that can cause oxidative damage to cellular structures such as lipids, proteins, and DNA and induce apoptosis [67,68].
Chlorin e6 (C6)	MACEDACEPhotoditazine λ = 660 nm	Chlorin-based PS (Derived from chlorophyll)	Type I and II	- Induce oxidative endoplasmic reticulum stress and DNA damage [69].
Bacteriochlorin (Ba)	TOOKAD λ = 753 or 762 nm	Chlorin-based PS (Derived from bacteriochlorophyll)	Type I and II	- ROS generation. - Blocks blood and nutriment supply to the vascular damage and tumor accumulation [70].
Pyropheophorbide (Py)	Pyropheophorbide-a methylester λ = 675 nm	Chlorin-based PS (Derived from chlorophyll)	Type II	- Targeting prostate-specific membrane antigen (PSMA) [71], - ROS generation and apoptosis induction via cytochrome c release, caspase 3 and caspase 9 activation [72].

* Depending on the solvent used for the measurement [73].

**Table 2:** An update of clinical studies conducted around the world to treat prostate cancer using PDT and combination approaches.

Intervention/ Treatment	Phase	Actual Enrollment	Identifier	Responsible Party	Results
Drug: 1 -mediated -VTP WST 1	Phase 1 Phase 2 Terminated	30	NCT00946881	UCLA—Jonsson Comprehensive Cancer Center Los Angeles, California, United States Midtown Urology & Midtown Urology Surgical Center Atlanta, Georgia, United States Washington University School of Medicine- Barnes-Jewish Hospital Saint Louis, Missouri, United States NYU Urology Associates, New York, New York, United States Memorial Sloan-Kettering Cancer Center, New York, New York, United States	The study showed that hemi-ablation of the prostate with WST11 vascular targeted PDT could be an effective treatment for prostate cancer. Optimal dosing parameters and light dose index were found to play important roles in the tissue response, as determined by MRI and biopsy [77].
Drug: WST09	Phase 2 Completed	28	NCT00308919	Princess Margaret Hospital Toronto, Ontario, Canada	No Results Posted
Drug: WST09 Vascular Photodynamic therapy	Phase 2 Phase 3 Terminated	16	NCT00312442	Abramson Cancer Center of The University of Pennsylvania Philadelphia, Pennsylvania, United States	No Results Posted
Drug: WST11	Phase 2 Completed	86	NCT00975429	Centre Hospitalier Universitaire (CHU) Angers, France Hôpital Claude Huriez Lille, France Institut Mutualiste Montsouris (IMM) Paris, France University College London Hospital (UCLH) Kings College Hospital (KCH) Frimley Park Hospital NHS Trust Catharina Ziekenhuis	No Results Posted
Drug: Motexafin lutetium	Phase 1 Terminated	24	NCT00005067	Abramson Cancer Center of The University of Pennsylvania Philadelphia, Pennsylvania, United States	No Results Posted
Drug: TOOKAD^®^ Soluble 4 mg/kg	Phase 2 Active	50	NCT03315754	Memorial Sloan-Kettering Cancer Center New York, New York, United States	No Results Posted
Focal therapies including PDT (PS undetermined)	Recruiting	200	NCT03492424	Weill Cornell Medicine New York, New York, United States	No Results Posted
Drug: WST11	Phase 2 Completed	42	NCT00707356	University Health Network-Princess Margaret Hospital Toronto, Canada Centre Hospitalier Universitaire Angers, France Hôpital Bocage-CHU Dijon, France Hôpital Claude Huriez, Lille, France Institut Mutualiste Montsouris (IMM), Paris, France Frimley Park Hospital NHS Trust, Frimley, United Kingdom	No Results Posted
Drug: TOOKAD^®^ Soluble	Phase 2 Completed	8	NCT00305929	The Prostate Centre Princess Margaret Hospital Toronto, Ontario, Canada	No Results Posted
Drug: TOOKAD^®^ Soluble	Phase 3 Completed	81	NCT01875393	Hospital General Tlahuac Mexico DF, Mexico Pan-American Medical Research Institute (PAMRI) then moved to Consultario del Dr Rodriguez Panama city, Panama Hospital Nacional Cayetano Heredia San Martin de Porres, Peru	No Results Posted
Drug: TOOKAD^®^ Soluble	Phase 3 Withdrawn	0	NCT04225299	Memorial Sloan-Kettering Cancer Center New York, New York, United States	No Results Posted
Drug: TOOKAD^®^ Soluble	Phase 4 Terminated	23	NCT03849365	Centre Hospitalier Universitaire Angers, France	No Results Posted
Drug: Visudyne^®^ Device: SpectraCure P18 System	Phase 1 Phase 2 Recruiting	66	NCT03067051	Memorial Sloan Kettering Cancer Center New York, New York, United States Keith Cengel Philadelphia, Pennsylvania, United States Princess Margaret Cancer Centre Toronto, Ontario, Canada	No Results Posted
Drug: TOOKAD^®^ Soluble	Phase 3 Completed	413	NCT01310894	Dept. of Urology-University Hospitals Leuven Leuven, Belgium Department of Urology-Tampere University Hospital- Tampere, Finland Service d’Urologie—Centre Hospitalier Universitaire Angers, France	No Results Posted

## Data Availability

Not applicable.
